# In vivo cortical diffusion imaging relates to Alzheimer’s disease neuropathology

**DOI:** 10.1186/s13195-023-01309-3

**Published:** 2023-10-04

**Authors:** Mario Torso, Gerard R. Ridgway, Michele Valotti, Ian Hardingham, Steven A. Chance, for the National Alzheimer’s Coordinating Center, James Brewer, Oscar Lopez, Bradley Hyman, Thomas Grabowski, Mary Sano, Helena Chui, Marilyn Albert, John Morris, Jeffrey Kaye, Thomas Wisniewski, Scott Small, John Trojanowski, Charles DeCarli, Andrew Saykin, David Bennett, Roger Rosenberg, Neil Kowall, Robert Vassar, Frank LaFerla, Ronald Petersen, Eric Reiman, Bruce Miller, Allan Levey, Linda Van Eldik, Sanjay Asthana, Russell Swerdlow, Todd Golde, Stephen Strittmatter, Victor Henderson, Suzanne Craft, Henry Paulson, Sudha Seshadri, Erik Roberson, Marwan Sabbagh, Gary Rosenberg, Angela Jefferson, Heather Whitson, James Leverenz, the Alzheimer’s Disease Neuroimaging Initiative

**Affiliations:** Oxford Brain Diagnostics Ltd, Oxford, UK

**Keywords:** Cortex, Minicolumns, Alzheimer’s disease neuropathological changes, Diffusion tensor imaging, Autopsy, Cortical diffusivity

## Abstract

**Background:**

There has been increasing interest in cortical microstructure as a complementary and earlier measure of neurodegeneration than macrostructural atrophy, but few papers have related cortical diffusion imaging to post-mortem neuropathology.

This study aimed to characterise the associations between the main Alzheimer’s disease (AD) neuropathological hallmarks and multiple cortical microstructural measures from in vivo diffusion MRI. Comorbidities and co-pathologies were also investigated.

**Methods:**

Forty-three autopsy cases (8 cognitively normal, 9 mild cognitive impairment, 26 AD) from the National Alzheimer’s Coordinating Center and Alzheimer’s Disease Neuroimaging Initiative databases were included. Structural and diffusion MRI scans were analysed to calculate cortical minicolumn-related measures (AngleR, PerpPD^+^, and ParlPD) and mean diffusivity (MD). Neuropathological hallmarks comprised Thal phase, Braak stage, neuritic plaques, and combined AD neuropathological changes (ADNC—the “ABC score” from NIA-AA recommendations).

Regarding comorbidities, relationships between cortical microstructure and severity of white matter rarefaction (WMr), cerebral amyloid angiopathy (CAA), atherosclerosis of the circle of Willis (ACW), and locus coeruleus hypopigmentation (LCh) were investigated.

Finally, the effect of coexistent pathologies—Lewy body disease and TAR DNA-binding protein 43 (TDP-43)—on cortical microstructure was assessed.

**Results:**

Cortical diffusivity measures were significantly associated with Thal phase, Braak stage, ADNC, and LCh. Thal phase was associated with AngleR in temporal areas, while Braak stage was associated with PerpPD^+^ in a wide cortical pattern, involving mainly temporal and limbic areas. A similar association was found between ADNC (ABC score) and PerpPD^+^.

LCh was associated with PerpPD^+^, ParlPD, and MD.

Co-existent neuropathologies of Lewy body disease and TDP-43 exhibited significantly reduced AngleR and MD compared to ADNC cases without co-pathology.

**Conclusions:**

Cortical microstructural diffusion MRI is sensitive to AD neuropathology. The associations with the LCh suggest that cortical diffusion measures may indirectly reflect the severity of locus coeruleus neuron loss, perhaps mediated by the severity of microglial activation and tau spreading across the brain.

Recognizing the impact of co-pathologies is important for diagnostic and therapeutic decision-making.

Microstructural markers of neurodegeneration, sensitive to the range of histopathological features of amyloid, tau, and monoamine pathology, offer a more complete picture of cortical changes across AD than conventional structural atrophy.

**Supplementary Information:**

The online version contains supplementary material available at 10.1186/s13195-023-01309-3.

## Introduction

The National Institute on Aging—Alzheimer’s Association (NIA-AA) guidelines for the neuropathological evaluation of Alzheimer’s disease (AD), consider extracellular amyloid-β (Aβ) plaques and intracellular neurofibrillary tangles (NFTs) of tau essential for the neuropathologic diagnosis of AD [1]. Previous studies [2, 3] showed these hallmarks of AD coincide with significant microstructural changes in cortical architecture and altered cellular minicolumnar organisation [2]. Extracellular Aβ plaques contribute to synaptic dysfunction [4] while progressive intracellular accumulation of NFTs leads to neuronal death [5].

Cortical minicolumns are narrow, vertical arrays of densely interconnected neurons spanning all cortical layers and representing a basic computational unit common to all cortical areas [6]. In some key AD regions, such as the parahippocampal gyrus, minicolumns become steadily thinner with the accretion of AD pathology and increase of plaque load [3]. Minicolumn thinning appears to precede minicolumn breakdown, and analysis of minicolumn structure in amnestic mild cognitive impairment (MCI) positions it between controls and AD [2]. Progressive minicolumnar structural alterations presumably contribute to functional disruption across multiple cognitive domains, and minicolumn width correlates with declining mini-mental state examination score across MCI and dementia [3]. However, in the early stages, cognitive deficits may be offset by ‘neural reserve’ wherein brain networks can absorb a degree of damage without noticeable effects or by active ‘neural compensation’ with damaged networks supplemented by recruiting additional resources [7].

A two-stage model has been proposed to describe cortical cytoarchitectural changes: a phase of synaptic and neuropil loss, minicolumn width decline with age, amyloid build up, and neuroinflammatory response, followed by a phase when cell loss and columnar disintegration occurs, and overt dementia develops [8].

The primary aim of the present study was to investigate associations between major Alzheimer’s neuropathological hallmarks and a set of cortical microstructural measures that relate to minicolumn structure, which have been validated using ex vivo [9] and in vivo cohorts [10–14].

AD pathology can exist in a “pure” form or co-exist with non-AD neuropathological changes and non-cortical neuropathological features that deserve to be investigated. The cortical pathology in AD is so dramatic that it may obscure the arterial and subcortical changes that occur during AD progression. Cerebral amyloid angiopathy (CAA) that involves small arteries, as well as arterioles in grey and white matter, occurs frequently during the progression of AD, and it contributes to vascular pathologies and white matter rarefaction (WMr). Depigmentation and loss of noradrenergic locus coeruleus projection neurons are commonly observed in the early stages of AD [15] and appear to be associated with increased cortical Aβ plaque and NFT loads [16]. Therefore, a second aim of the study was to investigate potential relationships between cortical diffusivity measures and non-cortical neuropathologic features, such as locus coeruleus hypopigmentation (LCh), CAA, WMr, and atherosclerosis of the circle of Willis (ACW).

Finally, many clinicopathological studies of dementia [17, 18] demonstrate that Alzheimer’s disease is frequently associated with other age-related processes, most commonly Lewy body disease (LBD) and TAR DNA-binding protein 43 (TDP-43) pathology. The third aim of the study was therefore to investigate the impact of the co-occurrence of AD neuropathology and the most common comorbidities like LBD and TDP-43 inclusions on cortical microstructure.

## Methods

### Participants

The present study comprised forty-three participants, ranging from cognitively normal to severe dementia, having diffusion tensor imaging (DTI) scans and neuropathological data, from the National Alzheimer’s Coordinating Center (NACC; 34 participants) and the Alzheimer’s Disease Neuroimaging Initiative (ADNI; 9 participants).

The NACC database (www.alz.washington.edu) provides data from patients collected in the AD Research Centers (ADRCs) funded by the National Institute on Aging. This analysis used data from five ADRCs collected between 2009 and 2015 [19].

ADNI was launched in 2003 as a public–private partnership, led by principal investigator Michael W. Weiner, MD. The primary goal of ADNI has been to test whether serial magnetic resonance imaging (MRI), positron emission tomography (PET), other biological markers, and clinical and neuropsychological assessment can be combined to measure the progression of MCI and early AD. For more information, please see www.adni-info.org.

Inclusion was limited to participants who were cognitively normal (CN) or had a clinical AD-type presentation (AD, MCI) who had undergone MRI scanning (T1-weighted structural and DTI) in life and had a subsequent neuropathological assessment at autopsy. The interval between MRI scan and autopsy ranged from 104 to 3278 days (median 1497 days).

At the time of the scanning (the closest available acquisition to death), participants were clinically diagnosed as: 8 CN, 9 MCI, and 26 AD. All clinical and neuropathological data were obtained through the NACC and ADNI data export.

To characterize the cohort, the Mini-Mental State Examination (MMSE), APOE ε4, Geriatric Depression Rating Scale (GDS), and Clinical Dementia Rating scale (CDR) global and sum of boxes (CDR-SB) scores were used.

### Neuropathological assessment

#### Brain autopsies

Autopsies and neuropathological evaluations were conducted according to the NACC protocols [20]. All the autopsies were collected with a post-mortem interval between 2 and 52 h, and brain tissues were fixed in formalin. At the time of autopsies, the average age for the CN group was 81.7, for MCI, 84.2, and for AD, 78.9 years.

#### Cortical and non-cortical neuropathologic changes

Neuropathology data was obtained from standard NACC and ADNI neuropathology reporting forms. Consistency of data between NACC and ADNI is maintained by using the same NACC Neuropathology Data Form to report autopsied cases [20].

Consistent with the current NIA-AA recommendations [1], the neuropathological assessment of Alzheimer’s disease neuropathologic change (ADNC) included the Thal phases of anatomical distribution of amyloid deposits [21], the Braak stages for tau neurofibrillary pathology (none, I–II, III–IV, V–VI) [22, 23], and the Consortium to Establish a Registry for Alzheimer’s Disease (CERAD) scores of neuritic plaque densities (none, sparse, moderate, frequent) [24]. For each case, the ADNC was evaluated using an “ABC” score that combines three separate four-point scales: amyloid-β plaques (A) by Thal phase assessment, NFT stage using Braak staging (B), and neuritic plaque score by CERAD score (C). The combination of A, B, and C scores determines a descriptor of “Not”, “Low”, Intermediate”, or “High” AD neuropathologic change. “Intermediate” or “High” AD neuropathologic change is considered sufficient explanation for dementia [1].

All these scores were used as classical neuropathological hallmarks for the post-mortem definition of AD and to investigate the relationship with a set of cortical diffusivity measures.

To investigate the relationship between cortical diffusivity measures and non-cortical neuropathologic changes, neuropathologists’ ratings of the severity of CAA, WMr, ACW, and LCh (graded semi-quantitatively as none, mild, moderate, or severe) were used as additional pathological markers.

#### Coexistent pathologic features at autopsy

To assess coexistent neuropathological changes of other diseases, the presence/absence of Vascular pathology (VP), TDP-43, Lewy body (LB) pathology, Creutzfeldt-Jakob disease and other prion encephalopathies (CJD), progressive supranuclear palsy (PSP), corticobasal degeneration (CBD), Pick’s disease (PiD), hippocampal sclerosis (HS), and amyotrophic lateral sclerosis (ALS) were defined according to the NACC coding guide.

Cases were defined with LB co-pathology if Lewy bodies were detected in limbic and neocortical regions.

Cases were considered to have TDP-43 co-pathology if TDP-43 deposition was observed in the hippocampus and entorhinal/inferior temporal cortex.

### MRI analysis

#### MRI acquisition and image pre-processing

For each participant, the closest scanning session to death that included both ante-mortem diffusion tensor imaging and 3D T1-weighted structural images was used. Data was acquired from 3.0 T MRI scanners (Siemens and GE Medical Systems) at multiple centres. For more details about structural and diffusion acquisition protocols, see www.alz.washington.edu and https://adni.loni.usc.edu/methods/documents/mri-protocols/.

The T1-weighted anatomical images were automatically processed using the FreeSurfer software version 6.0 (https://surfer.nmr.mgh.harvard.edu/). This provided outputs containing estimates of cortical volume, hippocampal volume, cortical thickness, white matter volume, and white matter hypointensities volume. Left and right hippocampal volumes were averaged. To account for head size differences, all volumes were expressed as a fraction of the total intracranial volume, namely cortical volume fraction, bilateral hippocampal fraction, white matter fraction, and white matter hypointensities fraction.

DTI data were processed using the FMRIB software library, (FSL Version 6.0.1, FMRIB, Oxford, UK, http://www.fmrib.ox.ac.uk/fsl/). Data was corrected for eddy current distortions and head motion, and the diffusion tensor model at each voxel was fitted using DTIFIT. To control for the effect of head motion in DTI maps, a displacement index was calculated using an in-house script.

#### Cortical diffusivity analysis

Cortical diffusivity analysis was performed using a proprietary software tool (Cortical Disarray Measurement, CDM; patent WO2016162682A1). The software generates cortical profiles, i.e. lines across the cortex in a radial direction, replicating columnar organisation within the cortex [9, 10]. Values for the diffusion tensor derived metrics were averaged along the cortical profiles, across the entire grey matter mask.

Briefly, the metrics calculated were mean diffusivity (MD) and three measures relating to the components of diffusion: AngleR is the angle between the radial minicolumn axis and the principal diffusion direction (in radians); ParlPD is the principal diffusion component parallel with the radial minicolumns (× 10^–3^ mm^2^/s); and PerpPD^+^ combines the components perpendicular to the radial minicolumns (× 10^–3^ mm^2^/s). PerpPD^+^ used here (and in ref.14) is a variant of the earlier PerpPD used in Torso et al.; [10] PerpPD^+^ includes multiple components (secondary and tertiary) orthogonal to the cortical columnar profile.

All the cortical values were averaged to reduce the influence of noise in the DTI scans, effectively smoothing the data, and ensuring only directionality with some local coherence would dominate, guarding against the influence of random deflections from the radial direction.

Cortical region values were extracted from whole brain DTI maps. A single arithmetic mean value was calculated for each cortical region based on the Desikan-Killiany cortical atlas.

### Statistical analyses

Data were analysed using IBM SPSS Statistics version 26 (SPSS, Chicago, IL). One-way ANOVAs were used to compare main effects for clinical and demographic variables in the groups and the chi-square test for categorical variables.

Univariate general linear model analyses were used to investigate diagnostic group differences in cortical diffusion measurements, using the diagnostic group code as a fixed factor, adjusted for the interval (days) between MRI scan date and autopsy date, scanner manufacturer, *b*-value, age, sex, and presence of comorbidities. The MRI macrostructural measures were assessed using the same model, excluding the covariate ‘*b*-value.’

Normality of the whole-brain microstructural measures was tested using Shapiro–Wilk tests. The results revealed a normal distribution for AngleR (*W* = 0.971, *p*-value = 0.482), ParlPD (*W* = 0.962, *p*-value = 0.279), while non-normal distributions were found for PerpPD^+^ (*W* = 0.978, *p*-value = 0.001) and MD (*W* = 0.923, *p*-value = 0.020). Based on this outcome, to investigate the potential associations between cortical diffusivity measures and histological measures, a non-parametric partial Spearman’s rank correlation analysis was used, controlling for interval (days) between MRI scan date and autopsy date, scanner manufacturer, *b*-values, age, sex, and presence of comorbidities. All results reported remained significant after false discovery rate correction (FDR < 0.05) [25].

Regional associations were investigated using linear models, controlling for the same variables used in whole-brain analysis (interval (days) between MRI scan date and autopsy date, scanner manufacturer, *b*-value, age, sex, and presence of comorbidities). Regional results reported were FDR corrected over the set of Desikan-Killiany regions.

Potential regional differences between groups with and without comorbidities were investigated using the cortical diffusivity measures (AngleR, PerpPD^+^, ParlPD, and MD) as dependent variables, with clinical diagnosis and ADNC score as fixed factors, controlling for interval (days) between MRI scan date and autopsy date, scanner manufacturer, *b*-value, age, and sex. The uncorrected *p*-values and FDR corrected results were reported.

## Results

### Participants

Clinical and demographic data are summarized in Table 1. Diagnostic groups were comparable for age, sex, and education. The proportion of APOE ε4 carriers/non-carriers was significantly different between groups, with a higher percentage of APOE ε4 carriers in the AD group. As expected, there were significant differences between groups for MMSE, Global CDR, and CDR-SB scores with a greater degree of impairment in the AD group. No other differences were detected.

**Table 1:** Clinical and demographic characteristics of participants

	CN (*n* = 8)	MCI (*n* = 9)	AD (*n* = 26)
Age at scan, mean (SD)	77.1 (6.2)	80.3 (9.7)	75.5 (6.8)
Women, *N* (%)	3 (37.5)	1 (11.1)	10 (38.5)
Education, mean (SD) years	15.4 (3.1)	16.1 (2.7)	15.8 (2.7)
APOE ε4 carriers (%)	2 (25)	3 (33.3)	19 (73.1) ^a,b^
MMSE, mean (SD)	28.7 (1.2)	26.1 (2.8)	21.9 (4.6) ^a,b^
GDS, mean (SD)	0.9 (0.9)	3.6 (3.9)	2.9 (3.2)
Global CDR®, mean (SD)	0.0 (0)	0.8 (0.5) ^a^	1.0 (0.4) ^a^
CDR-SB, mean (SD)	0.0 (0)	3.8 (3.2) ^a^	5.7 (2.2) ^a,b^
MRI scan/autopsy interval, mean (SD) days	1508.6 (967.6)	1368.2 (879.9)	1531.9 (884.3)
Diagnostic changes by 36 months	3 (to MCI)	8 (to AD)	0

ANOVA was used to identify differences between diagnostic groups

*APOE* apolipoprotein E, *MMSE* Mini-Mental State Examination, *GDS* Geriatric Depression Rating Scale, *CDR* Clinical Dementia Rating scale global, *CDR-SB* Clinical Dementia Rating scale sum of boxes (available only for NACC participants)

^a^Significant difference from CN

^b^Significant difference from MCI

### Neuropathological features

#### Alzheimer’s disease neuropathologic changes (ADNC) and clinical diagnosis

The ADNC severity, assessed following the NIA-AA recommendations, is reported for each case in Additional file 1: Table S3. The ADNC prevalence (calculated as the number of participants with the ADNC pathology/total autopsied participants) was 97.7% (42 cases). Out of the 43 cases included in the study, 35 (81.4%) had intermediate/high ADNC.

Divided by clinical diagnosis at the scan, in the CN group (8 cases), 1 case was “not AD”, 4 low ADNC, and 3 intermediate ADNC. Two of the three cases with intermediate ADNC converted to MCI by 36 months from the scan date.

In the MCI group (9 cases), 2 cases had low ADNC, 2 cases intermediate ADNC, and 5 cases high ADNC. Eight of the 9 MCI cases converted to AD by 36 months from the scan.

In the AD group (26 cases), 1 case showed low ADNC, 4 intermediate ADNC, and 21 high ADNC. No changes in clinical diagnosis were reported for this group.

#### Non-cortical neuropathologic changes

As summarized in Additional file 1: Table S4 (see also Additional file 1: Figure A1), non-cortical changes are reported frequently during the course of AD. The only case without ADNC showed a severe WMr, a moderate ACW, and a mild LCh. In cases with ADNC, the co-occurrence of non-cortical changes was slightly more common in cases with High ADNC. Out of 26 cases with high ADNC, a moderate/severe WMr was reported in 10 cases, a moderate/severe ACW in 8 cases, a moderate/severe LCh in 16 cases, and a moderate/severe CAA in 11 cases.

#### Coexistent pathologic features at autopsy

The pathologies coexistent with AD for each case included in the study are provided in Additional file 1: Table S5. As previously described, out of 43 cases, just one cognitively normal case was “Not-AD”. This case presented just moderate vascular signs and did not have any other pathology.

Out of the remaining 42 cases with AD neuropathological changes, 41 (97.6%) had at least one vascular sign, 14 (33.3%) were TDP-43 positive, 13 (30.9%) were LB positive, 1 (2.4%) had PiD, and 6 (14.3%) HS.

Considering the cases with at least 2 coexistent pathologies in addition to AD, 8 cases (19%) had VP and TDP-43, 8 (19%) VP and LB, and 2 (4.8%) VP and HS.

Three cases (7.1%) showed AD, VP, TDP-43, and LB; 1 case (2.4%) had AD, VP, TDP-43, and HS; and 1 case (2.4%) had AD, VP, LB, and HS.

One (2.4%) case showed AD, VP, TDP-43, PiD, and HS, and one case AD, VP, TDP-43, LB, and HS.

**Fig. 1:**
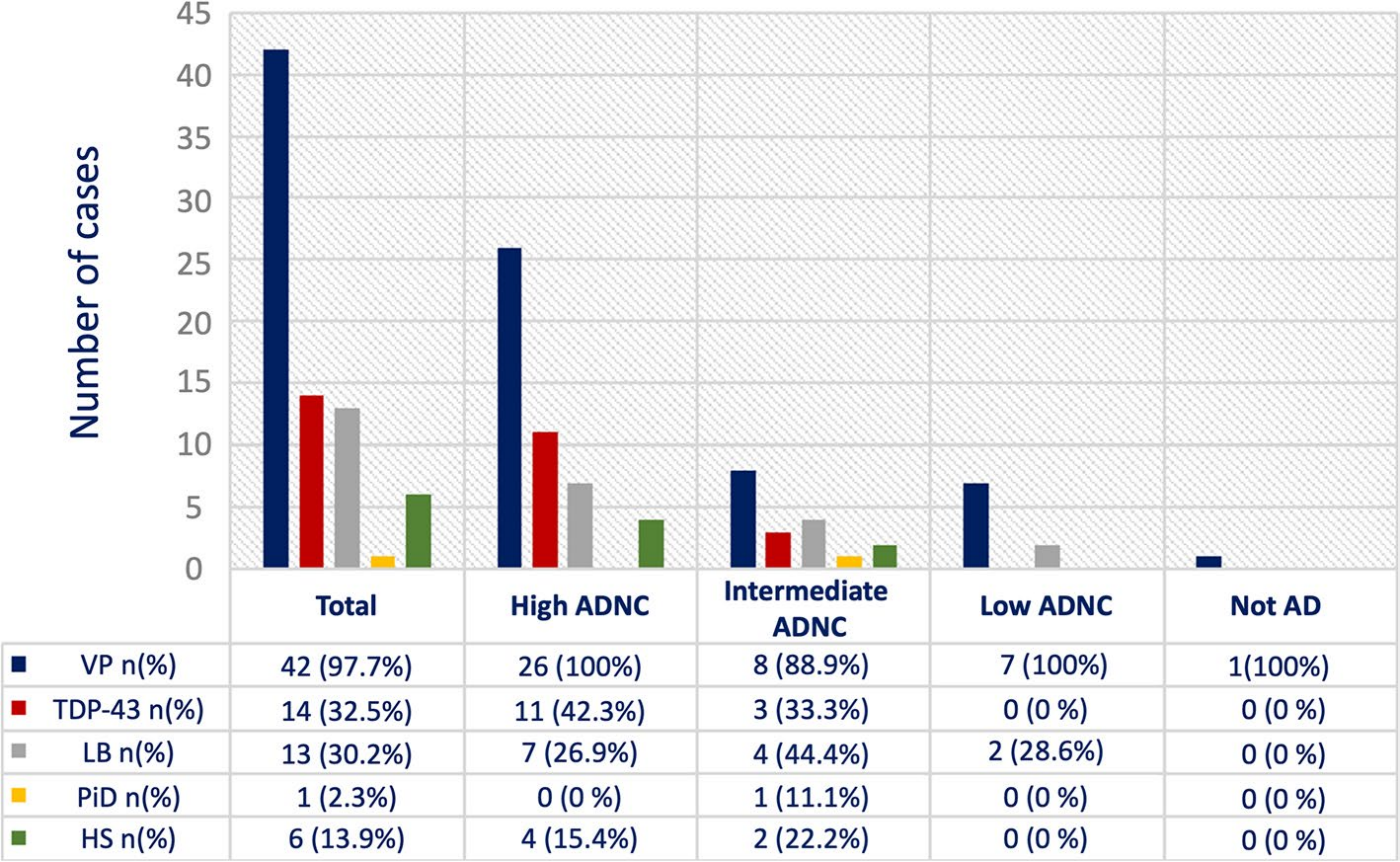
Number of cases with co-existent pathologies for each ADNC group. *VP* vascular pathology; *TDP-43* TAR DNA-binding protein 43 pathology; *LB* Lewy body disease; *PiD* Pick’s disease; *HS* hippocampal sclerosis

Grouping cases for ADNC severity, the high ADNC group had the highest number of coexistent pathologies (Fig. 1).

### MRI analysis

#### Macrostructural MRI

Based on the ADNC, participants were classified as “unlikely AD” (8 cases with ADNC of not or low) and “likely AD” (35 cases with ADNC of intermediate or high).

The two groups showed no significant differences in cortical thickness, white matter volume fraction, and white matter hypointensities fraction.

Significant differences after FDR correction between group means were found for cortical volume fraction (*F*_6,36_ = 9.21; *p* < 0.05; *η*_*p*_^2^ = 0.204) and bilateral hippocampal fraction (*F*_6,36_ = 7.14; *p* < 0.05; *η*_*p*_^2^ = 0.166).

#### “Microstructural” MRI: cortical diffusivity analysis

**Fig. 2:**
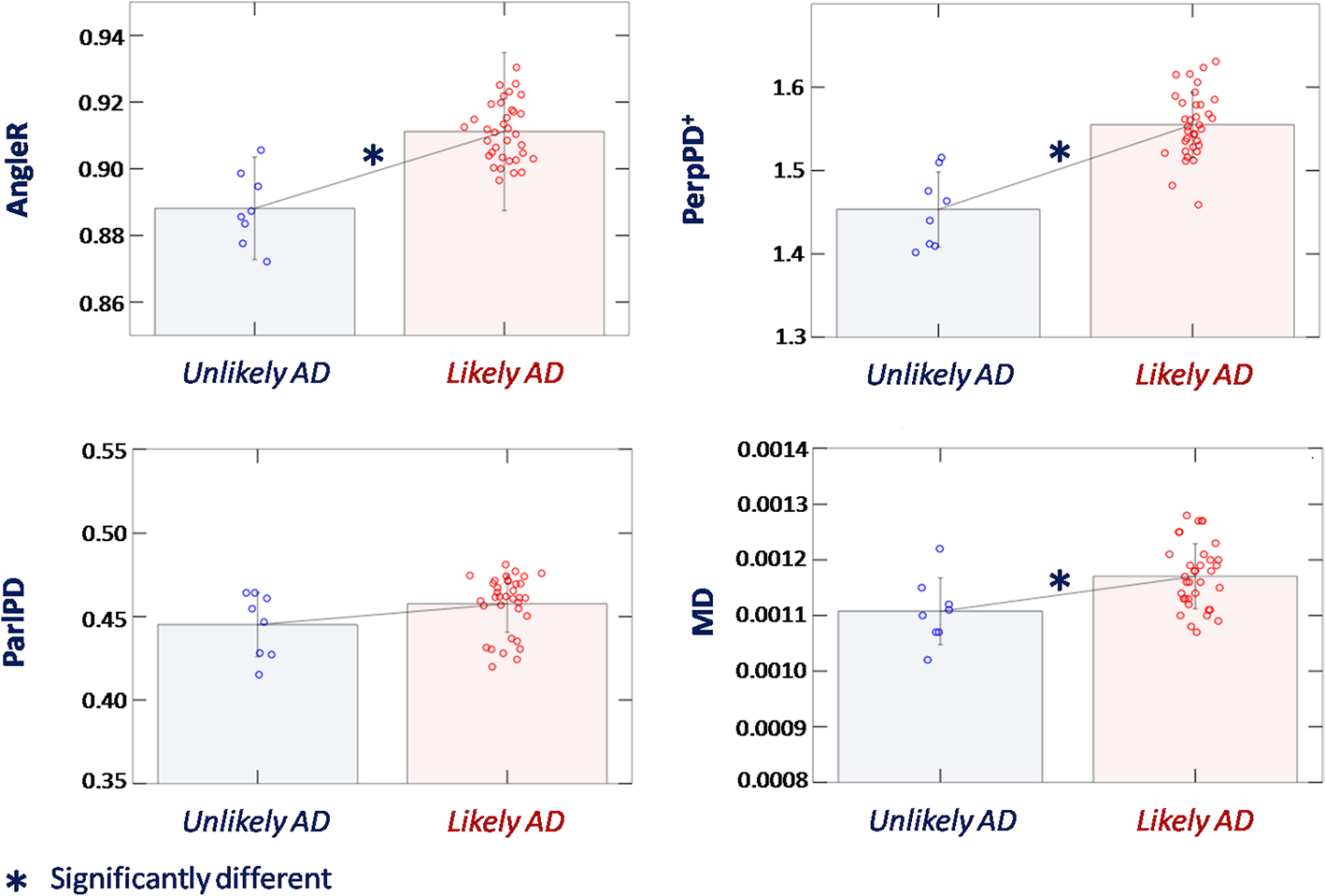
Whole brain cortical diffusivity differences between “unlikely AD” and “likely AD” groups. *Unlikely AD* ADNC not or low; *likely AD* ADNC of intermediate or high. Diagnostic groups were compared using a linear model. The group differences reported in the figure were significant after FDR correction

Comparison of the above-defined “likely AD” and “unlikely AD” groups revealed significant differences (after FDR correction) in AngleR (*F*_7,35_ = 6.25; *p* < 0.05; *η*_*p*_^2^ = 0.151), PerpPD^+^ (*F*_7,35_ = 5.64; *p* < 0.05; *η*_*p*_^2^ = 0.139), and MD (*F*_7,35_ = 4.87; *p* < 0.05; *η*_*p*_^2^ = 0.122) (Fig. 2). No significant differences between groups in ParlPD were detected.

#### Whole brain cortical diffusivity/neuropathology correlations

Two partial Spearman’s rank correlation analyses were performed to investigate associations between whole brain cortical diffusivity measures (AngleR, PerpPD^+^, ParlPD, and MD) and neuropathological scores.

**Fig. 3:**
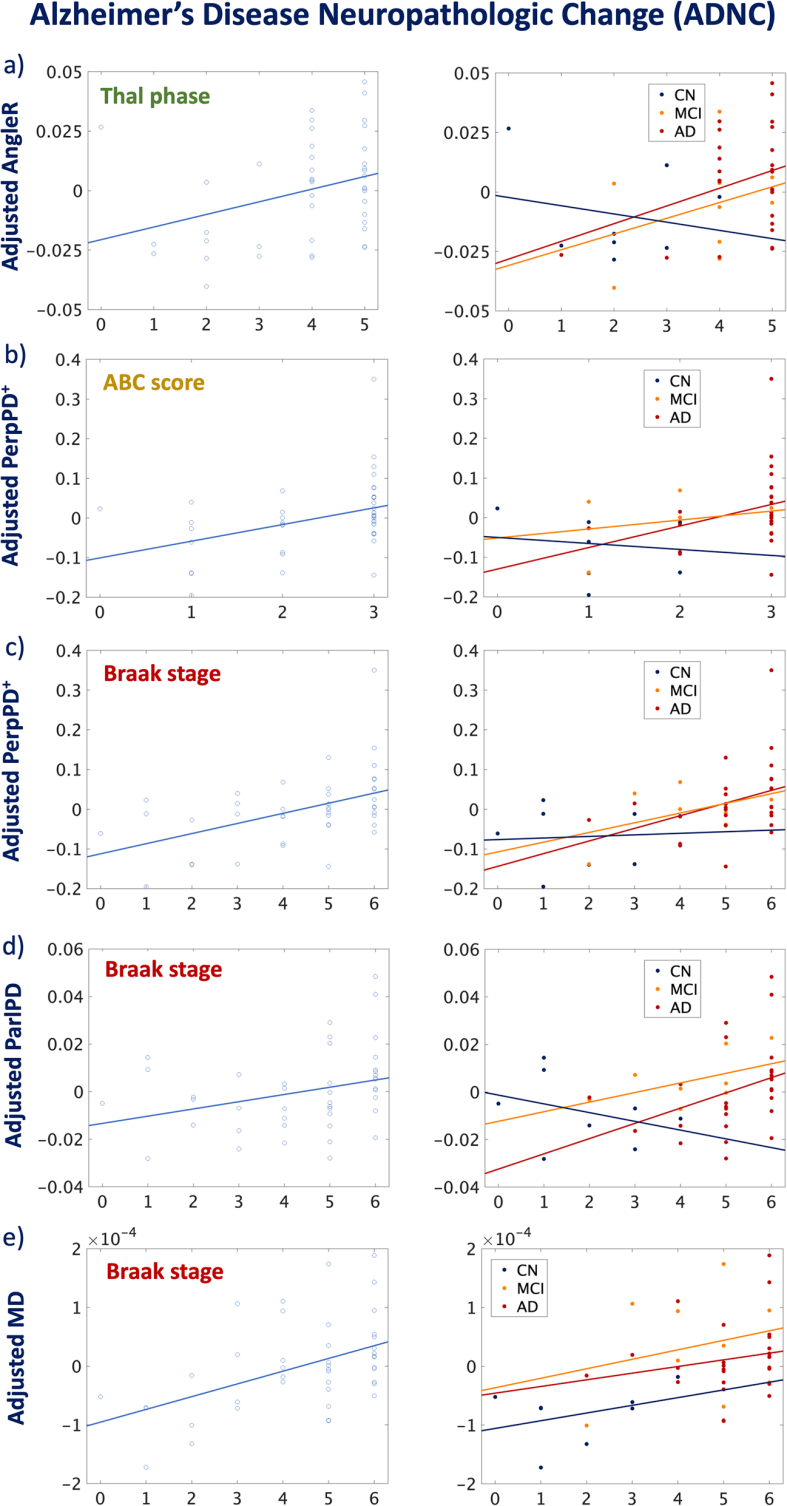
Significant correlations between cortical diffusivity metrics and ADNC. Thal phase (top row green box), ABC score (top row red box), and Braak stage (bottom row blue box). Colour codes indicate healthy controls (blue), individuals with MCI (orange), and AD cases (red). The whole sample correlation analysis revealed significant associations between cortical diffusivity metrics and ABC score (significant after FDR correction). The coloured lines for each group in AngleR, PerpPD^+^, and ParlPD values are consistent with the trajectory across the AD continuum [14], with a progressive increase in both patient groups (MD did not appear as sensitive to this aspect)

In the first analysis (Table 2), the association between cortical diffusivity measures and Alzheimer’s disease neuropathologic changes (Thal phase (A), Braak stage (B), and CERAD neuritic plaque (C) and ABC) scores was investigated. The results revealed significant positive associations (Fig. 3) between ADNC scores and cortical diffusivity measures (AngleR, PerpPD^+^, and MD).

**Table 2:** Associations between cortical diffusivity measures and AD neuropathology

	**Cortical neuropathology**			
	Thal phase (A)	Braak stage (B)	CERAD (C)	ABC
**AngleR**	ρ= 0.403; p= 0.013 pFDR= 0.041*	ρ= 0.342; p= 0.038 pFDR= n.s	ρ= 0.314; p= n.s pFDR= n.s	ρ= 0.330; p= 0.046 pFDR= n.s
**PerpPD**^**+**^	ρ= 0.247; p= n.s pFDR= n.s	ρ= 0.501; p= 0.002 pFDR= 0.032*	ρ= 0.186; p= n.s pFDR= n.s	ρ= 0.438; p= 0.007 pFDR= 0.037*
**ParlPD**	ρ= 0.099; p= n.s pFDR= n.s	ρ= 0.426; p= 0.009 pFDR= 0.036*	ρ= -0.028; p= n.s pFDR= n.s	ρ= 0.299; p= n.s pFDR= n.s
**MD**	ρ= 0.201; p= n.s pFDR= n.s	ρ= 0.448; p= 0.005 pFDR= 0.040*	ρ= 0.094; p= n.s pFDR= n.s	ρ= 0.357; p= 0.030 pFDR= n.s
	**Non-Cortical neuropathology**			
	**WMr**	**ACW**	**LCh**	**CAA**
**AngleR**	ρ**= -**0.130; p=n.s pFDR= n.s	ρ**= **0.998; p= n.s pFDR= n.s	ρ**= **0.450; p= 0.008 pFDR= 0.042*	ρ**= **0.215; p= n.s pFDR= n.s
**PerpPD**^**+**^	ρ**= **0.069; p= n.s pFDR= n.s	ρ**= **0.226; p= n.s pFDR= n.s	ρ**= **0.512; p= 0.002 pFDR= 0.024*	ρ**= **0.265; p= n.s pFDR= n.s
**ParlPD**	ρ**= **0.132; p= n.s pFDR= n.s	ρ**= **0.337; p= n.s pFDR= n.s	ρ**= **0.440; p= 0.009 pFDR= 0.036*	ρ**= **0.200; p= n.s pFDR= n.s
**MD**	ρ**= **0.081; p= n.s pFDR= n.s	ρ**= **0.176; p= n.s pFDR= n.s	ρ**= **0.491; p= 0.003 pFDR= 0.032*	ρ**= **0.190; p= n.s pFDR= n.s

*ρ* Spearman's rank partial correlation coefficient; *p* p value; *pFDR* Benjamini-Hochberg Adjusted *p* value. *WMr *white matter rarefaction; *ACW* atherosclerosis of the circle of Willis; *LCh* locus coeruleus hypopigmentation; *CAA *cerebral amyloid angiopathy

^*^Statistically significant difference after FDR correction

**Fig. 4:**
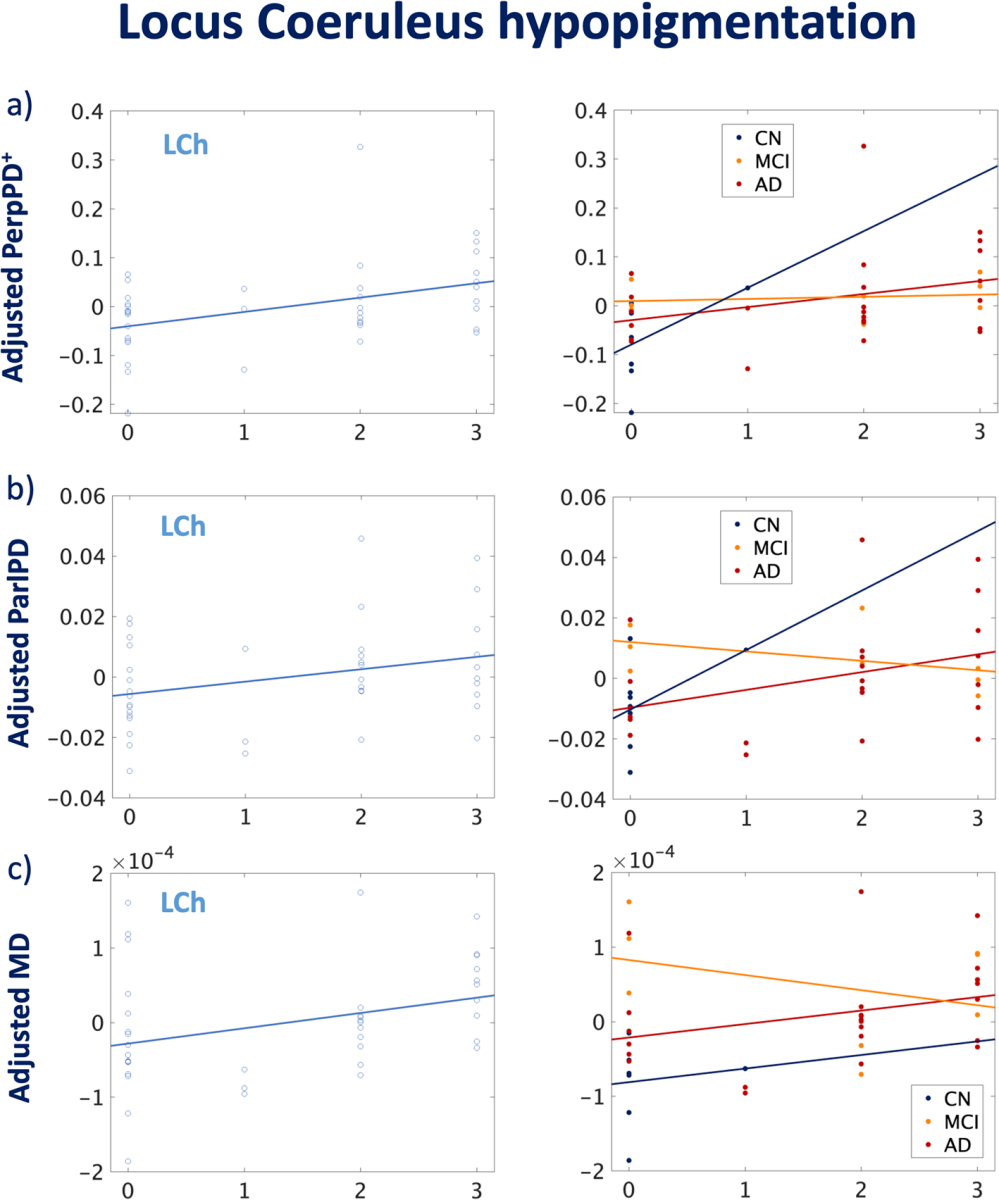
Correlation between cortical diffusivity measures and Locus Coeruleus hypopigmentation severity. Colour codes indicate healthy controls (blue), individuals with MCI (orange), and AD cases (red). All the associations reported in the figure were significant after FDR correction

In the second analysis, the association between cortical diffusivity measures and non-cortical neuropathologic changes (WMr, ACW, LCh, and CAA) was assessed. The LCh was the only non-cortical measure significantly associated with any (and all) cortical diffusivity measures (Table 2, Fig. 4).

#### Regional cortical diffusivity/neuropathology correlation

To further characterise the associations between cortical diffusivity measures and cortical AD neuropathologic changes that were seen at the whole brain level, the strongest associations reported (AngleR/Thal phase, PerpPD^+^/Braak stage, PerpPD^+^/ABC score) were explored at the regional level.

**Fig. 5:**
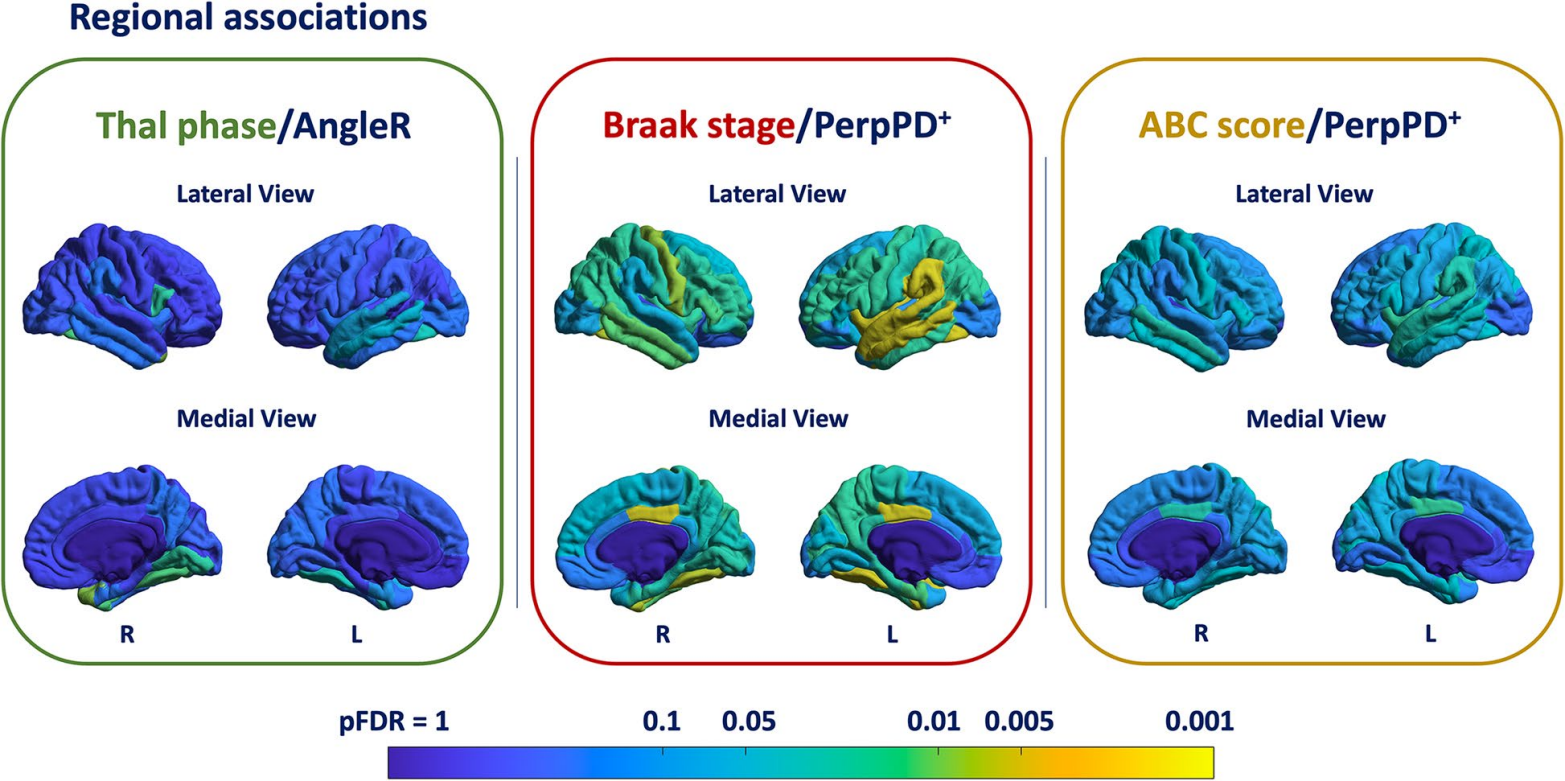
Cortical regions associated with ABC score. The colour bar shows the pFDR values. L, left hemisphere; R, right hemisphere

The results are summarized in Fig. 5. After FDR correction, the results showed significant associations between Thal phase score and AngleR values in different cortical regions, including the bilateral fusiform, left entorhinal, left middle temporal, right lingual, right pars opercularis, and right temporal pole.

Concerning the association between Braak stage and PerpPD^+^ values, the results clearly demonstrated a widespread significant association across cortical regions, involving, in particular, temporal (bilateral entorhinal, inferior temporal, middle temporal and fusiform cortex) and limbic regions (bilateral posterior cingulate cortex).

Finally, the regional analysis showed a significant pattern of association between PerpPD^+^ and the ABC score in temporal and parietal regions.

#### Coexistent pathologic features

**Fig. 6:**
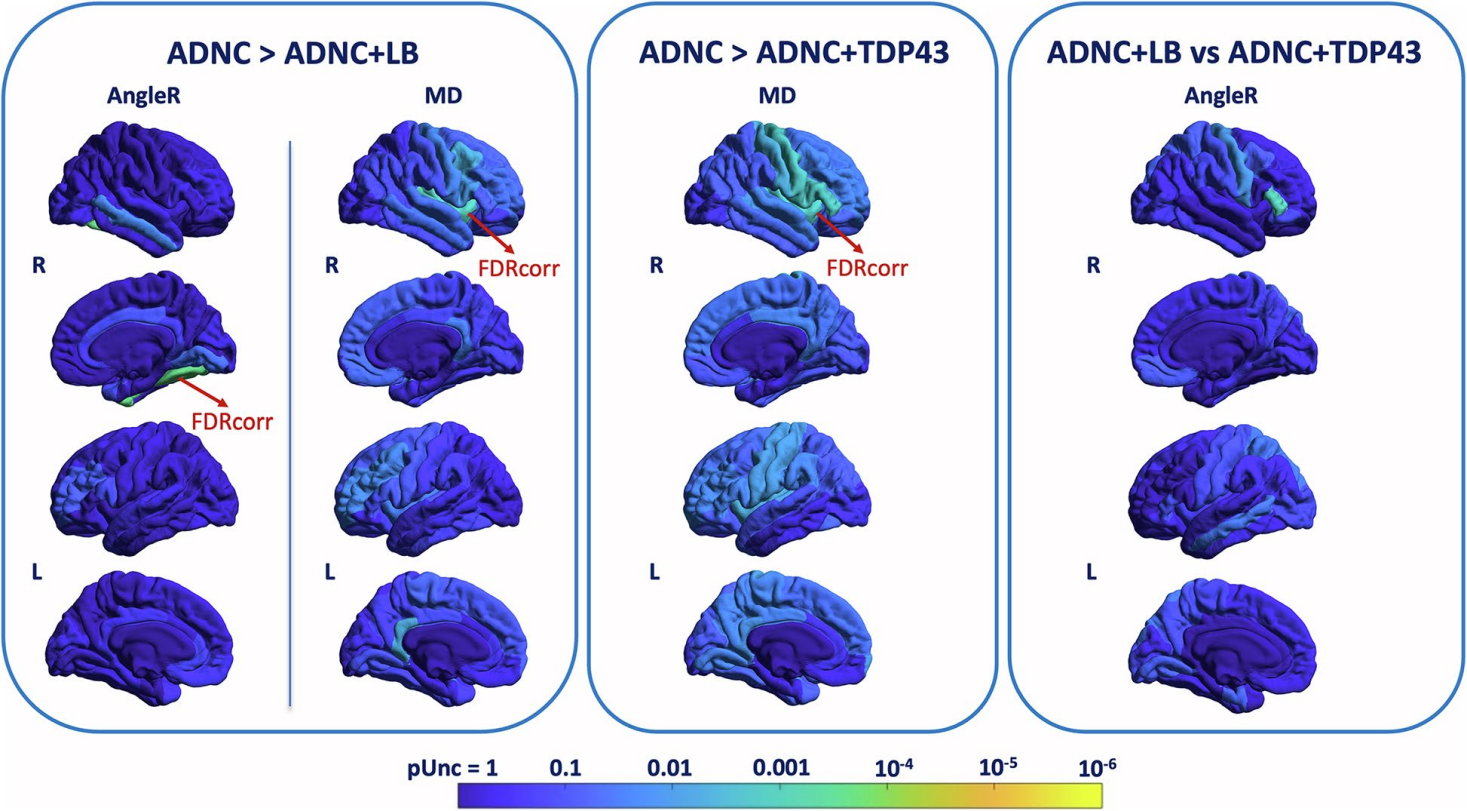
The impact of coexistent pathologic features on cortical diffusion values. The results of regional analysis performed using the cortical diffusivity measures (AngleR, PerpPD^+^, ParlPD, and MD) as dependent variables, adjusting for clinical diagnosis, ABC score, interval (days) between MRI scan date and autopsy date, scanner manufacturer, *b*-values, age, and sex. The figure shows uncorrected *p*-values. The red arrows indicate regions that remained significant after FDR correction (see the text for more details)

The cases with ADNC were grouped based on the presence/absence of additional pathologies (ADNC without LB or TDP-43, ADNC with LB and TDP-43, ADNC with LB only, and ADNC TDP-43 inclusions only), to explore the impact of coexistent pathology on cortical diffusivity measures. The results are shown in Fig. 6. At the uncorrected level, group comparisons showed that the cases with only ADNC compared to those with LB pathology had higher AngleR values in the right inferior temporal, lingual, and fusiform cortex and higher MD values in the bilateral caudal middle frontal, bilateral isthmus cingulate, right medial orbito-frontal, right middle temporal, right paracentral, right pars opercularis, right precentral, bilateral rostral middle frontal, right superior temporal, right transverse temporal, and bilateral insula. Differences in AngleR in the right fusiform, MD in the right transverse temporal, and right insula cortex remained significant after FDR correction.

Compared with the ADNC + TDP-43 group, at the uncorrected level, the ADNC only group showed a pattern of higher MD values that included the right banks STS, left isthmus cingulate, left lingual, left paracentral, right pars opercularis, right pars triangularis, bilateral postcentral, bilateral precentral, right posterior cingulate, bilateral rostral middle frontal, left supramarginal, left frontal pole, bilateral transverse temporal, and bilateral insula cortex. Differences in the right pars opercularis, right pars triangularis, right precentral, and right insula remained significant after FDR correction.

Finally, comparing the two groups with comorbidities, no significant differences were found after FDR correction. However, at the uncorrected level, the ADNC + LB group showed significant higher AngleR level in left middle temporal, left superior parietal, right pars triangularis, and right precentral cortex.

No significant differences were found in the ADNC group with both LB and TDP-43 comorbidities when compared to the ADNC group without comorbidities.

## Discussion

### AD neuropathological changes

Cortical diffusivity measures increase during AD progression, from “unlikely” to “likely” AD and are significantly associated with progression on Thal phase, Braak stage, and ABC score.

Findings were obtained from a cohort representative of the AD continuum. While, according to the amyloid cascade hypothesis [26], the role of Aβ protein in the pathogenesis of AD is crucial, the mere deposition of Aβ in the brain tissue is not sufficient to produce cognitive decline [27, 28]. AD progression can involve a long preclinical course of 15 − 25 years characterized by gradual accumulation of Aβ plaques across the cerebral cortex without any evidence of cognitive impairment [29]. Usually, cognitive decline occurs with neuritic and tau pathology, at an intermediate ADNC level, but about a third of people without dementia symptoms at autopsy have AD pathology meeting the criteria for intermediate ADNC [30, 31]. In the present study, only one CN case had ADNC of ‘not AD’, four had low ADNC, and three intermediate. Three out of eight CN cases converted to MCI within 36 months after the scan (two with intermediate ADNC, one with low ADNC); these cases presented only the coexistence of some vascular signs. All MCI participants with intermediate/high ADNC converted to AD by 36 months after scanning, while just one of the two MCI cases with low ADNC converted.

Clinical AD onset and progression can be mediated by many aspects, including genetic and epigenetic factors [32, 33], individual differences in susceptibility to age-related brain changes or AD-related pathology, (e.g. neural reserve, cognitive reserve) [34], and the co-occurrence of additional secondary neuropathological processes [18]. In addition, the modular organisation, connections, and plasticity of the cerebral cortex from minicolumns, to macrocolumns, to surface regions, could contribute to determining the pattern of pathological spread and, consequently, the pattern of functional loss [6].

The associations reported here between cortical diffusivity measures and AD neuropathological measures suggest that cortical diffusivity measures may be sensitive to alterations of minicolumnar microstructural organisation produced by AD neurodegeneration. A previous post-mortem validation study [9] demonstrated correlations between cortical diffusion measures and cortical minicolumnar architectural features, such as minicolumnar thinning.

Minicolumnar thinning has been reported to be associated with normal aging and to be more severe in dementia [2]. Previous studies [2, 3] showed that an increase of plaque load and thinning of the minicolumns continues steadily during AD progression [3]. This progressive minicolumn thinning appears to precede more severe minicolumn disruption due to loss of cell bodies and lost connections so that amnestic MCI looks neuropathologically intermediate between controls and AD [2]. The progressive increase of cortical diffusivity values during AD progression is therefore consistent with the trajectory of minicolumnar thinning and disruption that occurs during AD.

More specifically, as shown by the significant association with Thal phase, the AngleR metric seems sensitive to cortical microstructural changes determined by Aβ. These findings are consistent with previous in vivo evidence [14], which indicates that the AngleR metric may reflect cortical microstructural changes along the amyloid continuum, differentiating between amyloid positive and negative cognitively normal controls as well as distinguishing between amyloid positive controls and individuals with amyloid positive cognitive impairment (MCI and AD). Additionally, AngleR was found to be associated with markers of microglial activity, such as CSF soluble triggering receptor expressed on myeloid cells-2 (sTREM2) and progranulin (PGRN). Previous studies [14, 35] suggested that during AD progression, the trajectories of diffusion measures can be bi-phasic. Early Aβ deposition may cause an inflammatory response yielding a reduction of water diffusivity between minicolumns [14]. In the later stages, the progressive minicolumnar thinning and disruption due to neuronal, synaptic, and neuropil loss contribute to an increase of diffusivity between minicolumns.

Due to the small sample size of each diagnostic group in the present study, it was not possible to investigate the potential bi-phasic trajectory of diffusion measures along the AD continuum. However, consistent with previous studies [10, 14, 35], the results confirm a global increase in cortical diffusivity in the middle and late stages of AD. The significant associations between PerpPD^+^, ParlPD, and MD with Braak stage (Table 2 and Fig. 3) may reflect the progressive minicolumnar disruption due to neuronal loss processes like necroptosis [36]. Previous evidence [36, 37] suggested that tau accumulation could be a key trigger for necroptosis activation and the neurofibrillary tangles can represent an event proximal to neuronal loss in AD. The neuronal loss contributing strongly to minicolumnar disruption resulted in an increase of cortical diffusivity in all the main diffusion directions, especially in the axis perpendicular to minicolumns (PerpPD^+^).

In addition, the significant association between PerpPD^+^ and ABC scores suggests that PerpPD^+^ could be useful as a comprehensive indicator of neurodegeneration in the AD continuum.

### Locus coeruleus and noradrenergic system

The profound degeneration in noradrenergic neurons of the locus coeruleus is considered one of the earlier changes of AD [38]. This loss, and the resultant compensatory mechanisms in the remaining neurons, determine changes in the level of norepinephrine available in the brain [39].

As shown by previous studies, the locus coeruleus system plays a crucial protective role against brain diseases, improving the clearance of deposited Aβ and protecting against neuroinflammation and microglial activation and against tau pathology [40]. Activated microglia have a role in Aβ and tau spreading and in synapse loss [40]. The significant associations between cortical diffusion measures and locus coeruleus hypopigmentation suggest that cortical diffusion measures could reflect not just the severity of locus coeruleus neuron loss and lost projections indirectly, but in general, the severity of microglia activation and tau spreading across the brain that is associated with that cell loss.

### Coexistent pathologic features

Beyond ADNC, the most frequent coexistent proteinopathies in this cohort were TDP-43 and LB. The former involves primarily intraneuronal accumulations of phosphorylated TDP-43, while Lewy bodies are aggregates of phosphorylated a-synuclein in neuronal cytoplasmic inclusions [41]. As widely reported [42, 43], TDP-43 inclusions and Lewy body co-morbid proteinopathy frequently co-occur in patients with ADNC [44, 45]. However, it is not clear if the co-occurrence of LB and TDP-43 in AD is due to synergistic interactions or to overlapping of independent neuropathological processes.

In addition, TDP-43 is the main cause of a recently recognized disease entity, the limbic-predominant age-related TDP-43 encephalopathy (LATE) that seems to share some pathogenic mechanisms with both frontotemporal lobar degeneration with TDP-43 and Alzheimer’s disease, while also showing disease-specific underlying mechanisms [46].

To test whether the coexistence of ADNC and co-pathologies (LB and/or TDP-43) can produce specific changes in cortical diffusivity values, ADNC cases with and without comorbidities (LB and TDP-43) were compared. The cases were divided into four groups: ADNC without either LB or TDP-43, ADNC with LB, ADNC with TDP-43 inclusions, and ADNC with both LB and TDP-43.

Comparing groups, regional analyses revealed significant differences in AngleR and MD values (Fig. 6).

Although the small sample size demands prudence in interpretation, this significant difference represents an interesting aspect that can be better investigated in further studies to define the relationships between co-pathologies and their impact on clinical presentation and diagnostic process.

Compared with the ADNC only group, both groups with comorbidities showed lower AngleR and/or MD values. For the ADNC + LB group, the apparently ‘milder’ effect on cortical diffusivity values may be consistent with the findings of Buldyrev et al. [47] which showed that minicolumnar disruption was present in AD and LB but that the extensive loss of neurons (i.e. in the more advanced phase of progression in AD) was a feature of the AD brains, whereas the LB brains were relatively spared. The combination of coexisting LB and AD pathology on the progression of the columnar alterations may add further complexity, e.g. biphasic trajectory [14].

In neuropathological research, TDP-43 is classified into four subtypes (A, B, C, D). A recent study [48] demonstrated that there is distinct heterogeneity of TDP-43 deposition in non-FTLD brains, so the TDP-43 positive cases can show “typical” (type A) inclusions or TDP-43 immunoreactivity adjacent to/associated with a neurofibrillary tangle in the same neuron (type B).

In the cerebral cortex of patients with AD and dementia with Lewy bodies (DLB), type A is the most common [48, 49], and it is characterized by a large number of neuronal cytoplasmic inclusions and dystrophic neurites [48–50]. The dystrophic neurites may contribute to the reduction of AngleR values in subjects with coexistent ADNC + TDP-43 compared to ADNC only and ADNC + LB group.

### Limitations

The first limitation of the study is the sample size, which led us to restrict statistical analyses mainly to a whole brain level and a few regional level analyses. In particular, the sample size of each diagnostic group means that care must be taken not to over-interpret the separate regression lines for each group in Figs. 3 and 4. Furthermore, sample size limitations prevent us from carrying out statistical analysis of the small number of cases with HS (6) or PiD (1).

A second limitation arose from the significant time interval (approximately 4 years on average) between the last available imaging timepoint and the autopsy. A considerable number of subjects experienced a change in diagnostic categories within a span of 3 years. Consequently, some of the deductions drawn from imaging biomarkers may not assess directly all potential neuropathological changes that can occur some years later than the imaging.

An additional technical caveat as regards the MRI acquisition is that although all scans included here were acquired using broadly comparable protocols, and the scanner manufacturer and *b*-values were used as covariates in our analyses, it could be possible that correlation analyses may be more accurate using a dataset from a single centre.

## Conclusion

Taken together, these findings suggest that cortical diffusivity measures may reflect AD neuropathological changes in the microstructure of cortical grey matter. In vivo markers of neurodegeneration that are sensitive to the range of histopathological features of Aβ, tau, and monoamine pathology in Alzheimer’s disease offer a useful complement to existing non-invasive markers of amyloid and tau across the AD continuum.

In addition, further studies can better clarify if cortical diffusivity measures can help to investigate the frequent coexistence of AD proteinopathy (Aβ and tau) and other important age-related neuropathological features such as Lewy bodies, vascular disease, or TDP-43.

The identification of imaging patterns of comorbidities and their overlap and interaction may help to identify confounding factors in the diagnostic process and could help to target the development of early disease-modifying treatments.

### Supplementary Information

**Additional file 1: Table S3.** Individual ADNC scores in participants by clinical dementia status at scan. **Table S4.** Non-cortical neuropathological changes. **Table S5.** Coexistent pathologies. **Figure A1.** Co-occurrence of non-cortical changes. Co-pathology prevalence increases at higher levels of ADNC.

## Data Availability

The data supporting the conclusions of this article are available from the corresponding author on reasonable request. The datasets analysed during the current study are available in Alzheimer’s Disease Neuroimaging Initiative (ADNI) (http://adni.loni.usc.edu/) and National Alzheimer’s Coordinating Center (NACC) (http://www.alz.washington.edu).

## References

[1] Hyman BT, Phelps CH, Beach TG (2012). National Institute on Aging-Alzheimer’s Association guidelines for the neuropathologic assessment of Alzheimer’s disease. Alzheimer’s & dementia.

[2] Chance SA, Clover L, Cousijn H (2011). Microanatomical correlates of cognitive ability and decline: normal ageing, MCI, and Alzheimer’s disease. Cereb Cortex.

[3] van Veluw SJ, Sawyer EK, Clover L (2012). Prefrontal cortex cytoarchitecture in normal aging and Alzheimer’s disease: a relationship with IQ. Brain Struct Funct.

[4] Spires TL, Meyer-Luehmann M, Stern EA (2005). Dendritic spine abnormalities in amyloid precursor protein transgenic mice demonstrated by gene transfer and intravital multiphoton microscopy. J Neurosci.

[5] Gomez-Isla T, Hollister R, West H (1997). Neuronal loss correlates with but exceeds neurofibrillary tangles in Alzheimer’s disease. Ann Neurol.

[6] Casanova MF, Opris I (2015). Recent advances on the modular organization of the cortex.

[7] Stern Y (2009). Cognitive reserve. Neuropsychologia.

[8] Chance SA, Casanova MF, Switala AE (2006). Minicolumn thinning in temporal lobe association cortex but not primary auditory cortex in normal human ageing. Acta Neuropathol.

[9] McKavanagh R, Torso M, Jenkinson M (2019). Relating diffusion tensor imaging measurements to microstructural quantities in the cerebral cortex in multiple sclerosis. Hum Brain Mapp.

[10] Torso M, Bozzali M, Zamboni G, Jenkinson M, Chance SA (2021). Alzheimers Disease Neuroimage Initiative. Detection of Alzheimer’s disease using cortical diffusion tensor imaging. Human Brain Mapping..

[11] Torso M, Bozzali M, Cercignani M, Jenkinson M, Chance SA (2020). Using diffusion tensor imaging to detect cortical changes in fronto-temporal dementia subtypes. Sci Rep.

[12] Torso M, Ahmed S, Butler C, Zamboni G, Jenkinson M, Chance S (2021). Cortical diffusivity investigation in posterior cortical atrophy and typical Alzheimer’s disease. J Neurol.

[13] Torso M, Ridgway GR, Jenkinson M, Chance S (2021). Frontotemporal Lobar Degeneration Neuroimaging Initiative and the 4-Repeat Tau Neuroimaging Initiative (4RTNI). Intracortical diffusion tensor imaging signature of microstructural changes in frontotemporal lobar degeneration. Alzheimer’s Res Ther..

[14] Torso M, Ridgway GR, Hardingham I, et al. In vivo detection of changes related to cortical columnar organization and neuroinflammation across the AD continuum. J Prev Alzheimers Dis. 2022;9:769–79. 10.14283/jpad.2022.59. 10.14283/jpad.2022.59 36281682

[15] Kelly SC, He B, Perez SE, Ginsberg SD, Mufson EJ, Counts SE (2017). Locus coeruleus cellular and molecular pathology during the progression of Alzheimer’s disease. Acta Neuropathol Commun.

[16] Šimić G, Babić Leko M, Wray S (2017). Monoaminergic neuropathology in Alzheimer’s disease. Prog Neurobiol.

[17] Rabinovici GD, Carrillo MC, Forman M (2016). Multiple comorbid neuropathologies in the setting of Alzheimer’s disease neuropathology and implications for drug development. Alzheimers Dement.

[18] DeTure MA, Dickson DW (2019). The neuropathological diagnosis of Alzheimer’s disease. Mol Neurodegener.

[19] Besser L, Kukull W, Knopman DS (2018). Version 3 of the National Alzheimer’s Coordinating Center’s Uniform Data Set. Alzheimer Dis Assoc Disord.

[20] Besser LM, Kukull WA, Teylan MA (2018). The Revised National Alzheimer’s Coordinating Center’s Neuropathology Form-Available Data and New Analyses. J Neuropathol Exp Neurol.

[21] Thal DR, Rüb U, Orantes M, Braak H (2002). Phases of A beta-deposition in the human brain and its relevance for the development of AD. Neurology..

[22] Braak H, Braak E (1991). Neuropathological stageing of Alzheimer-related changes. Acta Neuropathol.

[23] Braak H, Alafuzoff I, Arzberger T, Kretzschmar H, Del Tredici K (2006). Staging of Alzheimer disease-associated neurofibrillary pathology using paraffin sections and immunocytochemistry. Acta Neuropathol.

[24] Mirra SS, Heyman A, McKeel D (1991). The Consortium to Establish a Registry for Alzheimer’s Disease (CERAD). Part II. Standardization of the neuropathologic assessment of Alzheimer’s disease. Neurology..

[25] Bejamini & Hochberg (1995). Journal of the Royal Statistical Society. Series B.

[26] Hardy JA, Higgings GA (1992). Alzheimer’s disease: the amyloid cascade hypothesis. Science.

[27] Aizenstein HJ, Nebes RD, Saxton JA (2008). Frequent amyloid deposition without significant cognitive impairment among the elderly. Arch Neurol.

[28] Villemagne VL, Pike KE, Chételat G (2011). Longitudinal assessment of Aβ and cognition in aging and Alzheimer disease. Ann Neurol.

[29] Jack CR, Knopman DS, Jagust WJ (2013). Tracking pathophysiological processes in Alzheimer’s disease: an updated hypothetical model of dynamic biomarkers. Lancet Neurol.

[30] Nelson PT, Braak H, Markesbery WR (2009). Neuropathology and cognitive impairment in Alzheimer disease: a complex but coherent relationship. J Neuropathol Exp Neurol.

[31] Bennett DA, Schneider JA, Arvanitakis Z, Kelly JF, Aggarwal NT, Shah RC, Wilson R (2006). Neuropathology of older persons without cognitive impairment from two community-based studies. Neurology.

[32] Lambert JC, Ibrahim-Verbaas CA, Harold D (2013). Meta-analysis of 74,046 individuals identifies 11 new susceptibility loci for Alzheimer’s disease. Nat Genet.

[33] Roubroeks JA, Smith RG, van den Hove DL, Lunnon K (2017). Epigenetics and DNA methylomic profiling in Alzheimer’s disease and other neurodegenerative diseases. J Neurochem.

[34] Stern Y (2012). Cognitive reserve in ageing and Alzheimer’s disease. The Lancet Neurology.

[35] Montal V, Vilaplana E, Alcolea D (2018). Cortical microstructural changes along the Alzheimer’s disease continuum. Alzheimer’s Dement.

[36] Caccamo A, Branca C, Piras IS (2017). Necroptosis activation in Alzheimer’s disease. Nat Neurosci.

[37] Nelson PT, Alafuzoff I, Bigio EH (2012). Correlation of Alzheimer disease neuropathologic changes with cognitive status: a review of the literature. J Neuropathol Exp Neurol.

[38] Braak H, Del Tredici K (2012). Where, when, and in what form does sporadic Alzheimer’s disease begin?. Curr Opin Neurol.

[39] Gannon M, Che P, Chen Y, Jiao K, Roberson ED, Wang Q (2015). Noradrenergic dysfunction in Alzheimer’s disease. Front Neurosci.

[40] Beardmore R, Hou R, Darekar A (2021). The locus coeruleus in aging and Alzheimer’s disease: a postmortem and brain imaging review. J Alzheimer’s Dis.

[41] Robinson JL, Richardson H, Xie SX (2021). The development and convergence of co-pathologies in Alzheimer’s disease. Brain.

[42] Spires-Jones TL, Attems J, Thal DR (2017). Interactions of pathological proteins in neurodegenerative diseases. Acta Neuropathol.

[43] Irwin DJ, Grossman M, Weintraub D (2017). Neuropathological and genetic correlates of survival and dementia onset in synucleinopathies: a retrospective analysis. Lancet Neurology.

[44] McAleese KE, Walker L, Erskine D, Thomas AJ, McKeith IG, Attems J (2017). TDP-43 pathology in Alzheimer’s disease, dementia with Lewy bodies and ageing. Brain Pathol.

[45] James BD, Wilson RS, Boyle PA, Trojanowski JQ, Bennett DA, Schneider JA (2016). TDP-43 stage, mixed pathologies, and clinical Alzheimer’s-type dementia. Brain.

[46] Nelson PT, Dickson DW, Trojanowski JQ (2019). Limbic-predominant age-related TDP-43 encephalopathy (LATE): consensus working group report. Brain..

[47] Buldyrev SV, Cruz L, Gomez-Isla T (2000). Description of microcolumnar ensembles in association cortex and their disruption in Alzheimer and Lewy body dementias. Proc Natl Acad Sci.

[48] Josephs KA, Murray ME, Tosakulwong N (2019). Pathological, imaging and genetic characteristics support the existence of distinct TDP-43 types in non-FTLD brains. Acta Neuropathol.

[49] Josephs KA, Murray ME, Whitwell JL (2016). Updated TDP-43 in Alzheimer’s disease staging scheme. Acta Neuropathol.

[50] Huang W, Zhou Y, Tu L (2020). TDP-43: from Alzheimer’s disease to limbic-predominant age-related TDP-43 encephalopathy. Frontiers Molecular Neuroscience.

