# Supplementary material for: In vivo cortical diffusion imaging relates to Alzheimer’s disease neuropathology
Source: Alzheimers Res Ther. 2023 Oct 4;15:165. doi: 10.1186/s13195-023-01309-3 (PMC10548768; doi:10.1186/s13195-023-01309-3)
Supplement: Supplementary file 1 — Additional file 1: Table S3. Individual ADNC scores in participants by clinical dementia status at scan. Table S4. Non-cortical neuropathological changes. Table S5. Coexistent pathologies. Figure A1. Co-occurrence of non-cortical changes. Co-pathology prevalence increases at higher levels of ADNC. [file 13195_2023_1309_MOESM1_ESM.docx]

**Additional file 1**

**Table 3 Individual ADNC scores in participants by clinical dementia status at scan**

| ID | *A* THAL | *B* BRAAK | *C*  CERAD | NIA-AA  ADNC (ABC score) | Clinical Diagnosis at scan | Diagnostic  Changes |
| --- | --- | --- | --- | --- | --- | --- |
| 1 | A2 | B1 | C0 | Low ADNC | CN | Stable |
| 2 | A0 | B1 | C0 | Not AD | CN | Stable |
| 3 | A3 | B3 | C3 | Intermediate ADNC | CN | Stable |
| 4 | A3 | B3 | C2 | Intermediate ADNC | CN | Converted to MCI |
| 5 | A1 | B1 | C0 | Low ADNC | CN | Stable |
| 6 | A4 | B4 | C3 | Intermediate ADNC | CN | Converted to MCI |
| 7 | A2 | B1 | C0 | Low ADNC | CN | Converted to MCI |
| 8 | A2 | B2 | C1 | Low ADNC | CN | Stable |
| 9 | A2 | B2 | C0 | Low ADNC | MCI | Converted to AD |
| 10 | A4 | B6 | C2 | High ADNC | MCI | Converted to AD |
| 11 | A5 | B4 | C3 | Intermediate ADNC | MCI | Converted to AD |
| 12 | A4 | B5 | C3 | High ADNC | MCI | Converted to AD |
| 13 | A5 | B5 | C2 | High ADNC | MCI | Converted to AD |
| 14 | A2 | B3 | C1 | Low ADNC | MCI | Stable |
| 15 | A4 | B6 | C3 | High ADNC | MCI | Converted to AD |
| 16 | A4 | B5 | C2 | High ADNC | MCI | Converted to AD |
| 17 | A4 | B4 | C2 | Intermediate ADNC | MCI | Converted to AD |
| 18 | A5 | B6 | C3 | High ADNC | AD | Stable |
| 19 | A5 | B5 | C3 | High ADNC | AD | Stable |
| 20 | A5 | B5 | C3 | High ADNC | AD | Stable |
| 21 | A4 | B5 | C3 | High ADNC | AD | Stable |
| 22 | A4 | B5 | C3 | High ADNC | AD | Stable |
| 23 | A5 | B5 | C3 | High ADNC | AD | Stable |
| 24 | A5 | B4 | C1 | Intermediate ADNC | AD | Stable |
| 25 | A5 | B6 | C3 | High ADNC | AD | Stable |
| 26 | A4 | B5 | C3 | High ADNC | AD | Stable |
| 27 | A5 | B6 | C3 | High ADNC | AD | Stable |
| 28 | A4 | B6 | C3 | High ADNC | AD | Stable |
| 29 | A1 | B2 | C0 | Low ADNC | AD | Stable |
| 30 | A5 | B6 | C3 | High ADNC | AD | Stable |
| 31 | A5 | B6 | C3 | High ADNC | AD | Stable |
| 32 | A4 | B5 | C3 | High ADNC | AD | Stable |
| 33 | A5 | B6 | C2 | High ADNC | AD | Stable |
| 34 | A5 | B6 | C3 | High ADNC | AD | Stable |
| 35 | A3 | B4 | C3 | Intermediate ADNC | AD | Stable |
| 36 | A4 | B6 | C2 | High ADNC | AD | Stable |
| 37 | A5 | B6 | C3 | High ADNC | AD | Stable |
| 38 | A5 | B6 | C3 | High ADNC | AD | Stable |
| 39 | A5 | B6 | C3 | High ADNC | AD | Stable |
| 40 | A5 | B5 | C3 | High ADNC | AD | Stable |
| 41 | A4 | B5 | C3 | High ADNC | AD | Stable |
| 42 | A5 | B4 | C2 | Intermediate ADNC | AD | Stable |
| 43 | A4 | B3 | C2 | Intermediate ADNC | AD | Stable |

THAL= Thal phase for amyloid plaques (A score); BRAAK= Braak stage for neurofibrillary degeneration (B score); CERAD= Density of neocortical neuritic plaques (CERAD score) (C score).

**Table 4 Non-cortical neuropathological changes**

| ID | CDS | ADNC | WMr | ACW | LCh | CAA |
| --- | --- | --- | --- | --- | --- | --- |
| 1 | CN | Low ADNC | None | None | None | Mild |
| 2 | CN | Not AD | Severe | Moderate | Mild | Mild |
| 3 | CN | Intermediate ADNC | Moderate | Moderate | None | Mild |
| 4 | CN | Intermediate ADNC | None | Severe | None | None |
| 5 | CN | Low ADNC | Moderate | Mild | None | None |
| 6 | CN | Intermediate ADNC | None | None | None | Mild |
| 7 | CN | Low ADNC | None | Mild | None | None |
| 8 | CN | Low ADNC | None | Severe | None | None |
| 9 | MCI | Low ADNC | None | - | - | Moderate |
| 10 | MCI | High ADNC | Moderate | Severe | Moderate | Severe |
| 11 | MCI | Intermediate ADNC | None | None | Severe | Mild |
| 12 | MCI | High ADNC | Moderate | Mild | Moderate | None |
| 13 | MCI | High ADNC | Mild | Moderate | None | None |
| 14 | MCI | Low ADNC | Moderate | Moderate | None | Mild |
| 15 | MCI | High ADNC | Moderate | Mild | Severe | Severe |
| 16 | MCI | High ADNC | Moderate | Moderate | None | Moderate |
| 17 | MCI | Intermediate ADNC | Moderate | Mild | Severe | None |
| 18 | AD | High ADNC | None | None | Severe | Severe |
| 19 | AD | High ADNC | None | Mild | None | Mild |
| 20 | AD | High ADNC | None | Mild | None | Mild |
| 21 | AD | High ADNC | None | Mild | None | Moderate |
| 22 | AD | High ADNC | None | None | None | Mild |
| 23 | AD | High ADNC | None | - | - | Moderate |
| 24 | AD | Intermediate ADNC | None | - | - | Mild |
| 25 | AD | High ADNC | None | None | None | Mild |
| 26 | AD | High ADNC | Mild | Mild | Severe | Mild |
| 27 | AD | High ADNC | Moderate | Mild | Moderate | Severe |
| 28 | AD | High ADNC | Moderate | Severe | Moderate | Mild |
| 29 | AD | Low ADNC | Mild | None | Moderate | None |
| 30 | AD | High ADNC | Mild | Mild | Moderate | Mild |
| 31 | AD | High ADNC | Mild | Severe | Severe | Mild |
| 32 | AD | High ADNC | Moderate | Moderate | Mild | Mild |
| 33 | AD | High ADNC | None | Moderate | Moderate | Moderate |
| 34 | AD | High ADNC | None | Mild | Severe | Mild |
| 35 | AD | Intermediate ADNC | Severe | Severe | None | Mild |
| 36 | AD | High ADNC | Moderate | Mild | Severe | Severe |
| 37 | AD | High ADNC | Mild | None | Severe | Severe |
| 38 | AD | High ADNC | Moderate | Severe | Severe | Severe |
| 39 | AD | High ADNC | None | Mild | Moderate | Mild |
| 40 | AD | High ADNC | Moderate | Mild | Mild | Mild |
| 41 | AD | High ADNC | None | Mild | Moderate | Mild |
| 42 | AD | Intermediate ADNC | Mild | Mild | Moderate | Mild |
| 43 | AD | Intermediate ADNC | None | None | Moderate | Moderate |

CDS= Clinical diagnosis at the scan; ADNC= Alzheimer's disease neuropathologic change (ADNC); WMr= white matter rarefaction; ACW= Atherosclerosis of circle of Willis; LCh= Locus Coeruleus hypopigmentation; CAA= Cerebral amyloid angiopathy.

**Figure A1**


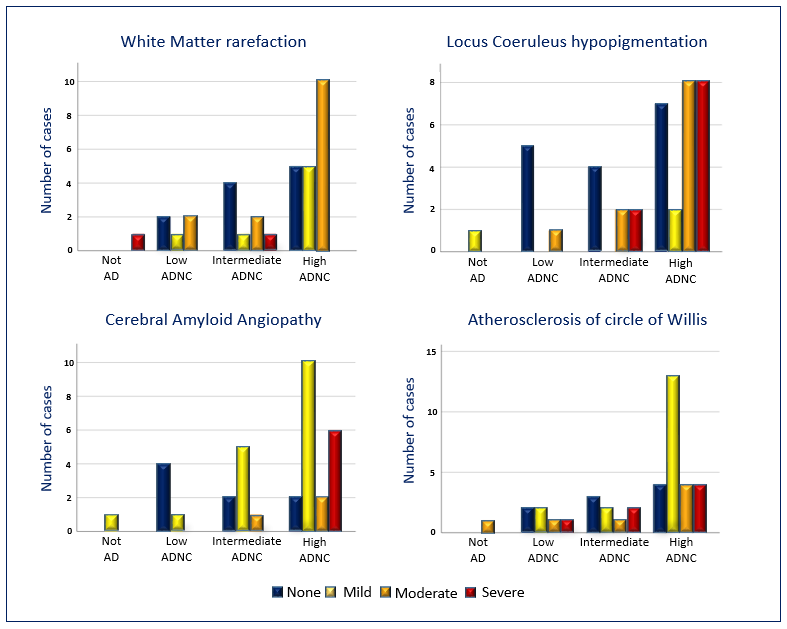


**Figure A1 Co-occurrence of non-cortical changes.** Co-pathology prevalence increases at higher levels of ADNC.

**Table 5 Coexistent pathologies**

| ID | CDS | ADNC | VP | TDP-43 | LB | CJD | PSP | CBD | PiD | HS | ALS |
| --- | --- | --- | --- | --- | --- | --- | --- | --- | --- | --- | --- |
| 1 | CN | Low ADNC | X | - | - | - | - | - | - | - | - |
| 2 | CN | Not AD | X | - | - | - | - | - | - | - | - |
| 3 | CN | Intermediate ADNC | - | - | - | - | - | - | - | - | - |
| 4 | CN | Intermediate ADNC | X | - | - | - | - | - | - | - | - |
| 5 | CN | Low ADNC | X | - | - | - | - | - | - | - | - |
| 6 | CN | Intermediate ADNC | X | - | - | - | - | - | - | - | - |
| 7 | CN | Low ADNC | X | - | - | - | - | - | - | - | - |
| 8 | CN | Low ADNC | X | - | - | - | - | - | - | - | - |
| 9 | MCI | Low ADNC | X | - | X | - | - | - | - | - | - |
| 10 | MCI | High ADNC | X | - | - | - | - | - | - | - | - |
| 11 | MCI | Intermediate ADNC | X | - | X | - | - | - | - | - | - |
| 12 | MCI | High ADNC | X | X | - | - | - | - | - | - | - |
| 13 | MCI | High ADNC | X | - | - | - | - | - | - | - | - |
| 14 | MCI | Low ADNC | X | - | - | - | - | - | - | - | - |
| 15 | MCI | High ADNC | X | - | X | - | - | - | - | X | - |
| 16 | MCI | High ADNC | X | - | - | - | - | - | - | X | - |
| 17 | MCI | Intermediate ADNC | X | X | - | - | - | - | X | X | - |
| 18 | AD | High ADNC | X | X | X | - | - | - | - | - | - |
| 19 | AD | High ADNC | X | - | X | - | - | - | - | - | - |
| 20 | AD | High ADNC | X | X | - | - | - | - | - | - | - |
| 21 | AD | High ADNC | X | - | - | - | - | - | - | - | - |
| 22 | AD | High ADNC | X | - | - | - | - | - | - | - | - |
| 23 | AD | High ADNC | X | - | X | - | - | - | - | - | - |
| 24 | AD | Intermediate ADNC | X | X | X | - | - | - | - | - | - |
| 25 | AD | High ADNC | X | - | X | - | - | - | - | - | - |
| 26 | AD | High ADNC | X | X | - | - | - | - | - | - | - |
| 27 | AD | High ADNC | X | - | X | - | - | - | - | - | - |
| 28 | AD | High ADNC | X | - | - | - | - | - | - | - | - |
| 29 | AD | Low ADNC | X | - | X | - | - | - | - | - | - |
| 30 | AD | High ADNC | X | - | - | - | - | - | - | - | - |
| 31 | AD | High ADNC | X | X | - | - | - | - | - | X | - |
| 32 | AD | High ADNC | X | X | - | - | - | - | - | - | - |
| 33 | AD | High ADNC | X | - | - | - | - | - | - | - | - |
| 34 | AD | High ADNC | X | X | - | - | - | - | - | - | - |
| 35 | AD | Intermediate ADNC | X | - | - | - | - | - | - | X | - |
| 36 | AD | High ADNC | X | - | - | - | - | - | - | - | - |
| 37 | AD | High ADNC | X | X | - | - | - | - | - | - | - |
| 38 | AD | High ADNC | X | X | X | - | - | - | - | X | - |
| 39 | AD | High ADNC | X | X | - | - | - | - | - | - | - |
| 40 | AD | High ADNC | X | X | - | - | - | - | - | - | - |
| 41 | AD | High ADNC | X | - | - | - | - | - | - | - | - |
| 42 | AD | Intermediate ADNC | X | X | X | - | - | - | - | - | - |
| 43 | AD | Intermediate ADNC | X | - | X | - | - | - | - | - | - |

CDS= Clinical diagnosis at the scan; AD= Histopathologically defined AD; VP= Vascular pathology; TDP-43= TAR DNA-binding protein 43 pathology; LB= Lewy body disease; CJD= Creutzfeldt-Jakob disease and other prion encephalopathies; PSP= Progressive supranuclear palsy; CBD= Corticobasal degeneration; PiD= Pick's disease; HS= Hippocampal sclerosis; ALS= Amyotrophic lateral sclerosis.
